# Supplementary material for: MacroD1 sustains mitochondrial integrity and oxidative metabolism
Source: Nat Commun. 2025 Aug 15;16:7595. doi: 10.1038/s41467-025-62410-9 (PMC12356970; doi:10.1038/s41467-025-62410-9)
Supplement: Supplementary file 1 — Supplementary Information [file 41467_2025_62410_MOESM1_ESM.pdf]

SUPPLEMENTARY INFORMATION

MacroD1 sustains mitochondrial integrity and oxidative metabolism

Ann-Katrin Hopp[1,2], Lorenza P. Ferretti[1], Lisa Schlicker[3], Amalia Ruiz-Serrano[4], Udo Hetzel[5], Francesco Prisco[6], Anja Kipar[6], Lukas Muskalla[1,2], Elena Ferrari[1], Carsten C. Scholz[4,7], Karsten Hiller[3], Francisco Verdeguer[1], Deena M. Leslie Pedrioli[1] and Michael O. Hottiger[1]*

[1] Department of Molecular Mechanisms of Disease (DMMD); University of Zurich; 8057 Zurich; Switzerland

[2] Life Science Zurich Graduate School; Molecular Life Science Ph.D. Program; Zurich; Switzerland

[3] Institute for Biochemistry, Biotechnology and Bioinformatics, Braunschweig Integrated Centre of Systems Biology (BRICS), 38106 Braunschweig; Germany

[4] Institute of Physiology; University of Zurich; 8057 Zurich; Switzerland

[5] Electron Microscopy Unit, Institute of Veterinary Pathology, University of Zurich, 8057 Zurich; Switzerland

[6] Laboratory for Animal Model Pathology (LAMP), Institute of Veterinary Pathology, University of Zurich, 8057 Zurich; Switzerland

[7] Institute of Physiology, University Medicine Greifswald, 17475 Greifswald, Germany

* Correspondence to: michael.hottiger@dmmd.uzh.ch.

List of Supplementary Figures and Files:

Suppl. Fig. 1
Suppl. Fig. 2
Suppl. Fig. 3
Suppl. Fig. 4
Suppl. Fig. 5
Suppl. Fig. 6
Suppl. Fig. 7
Suppl. Fig. 8

Suppl. Data 1
Suppl. Data 2
Suppl. Data 3

SUPPLEMENTARY FIGURES

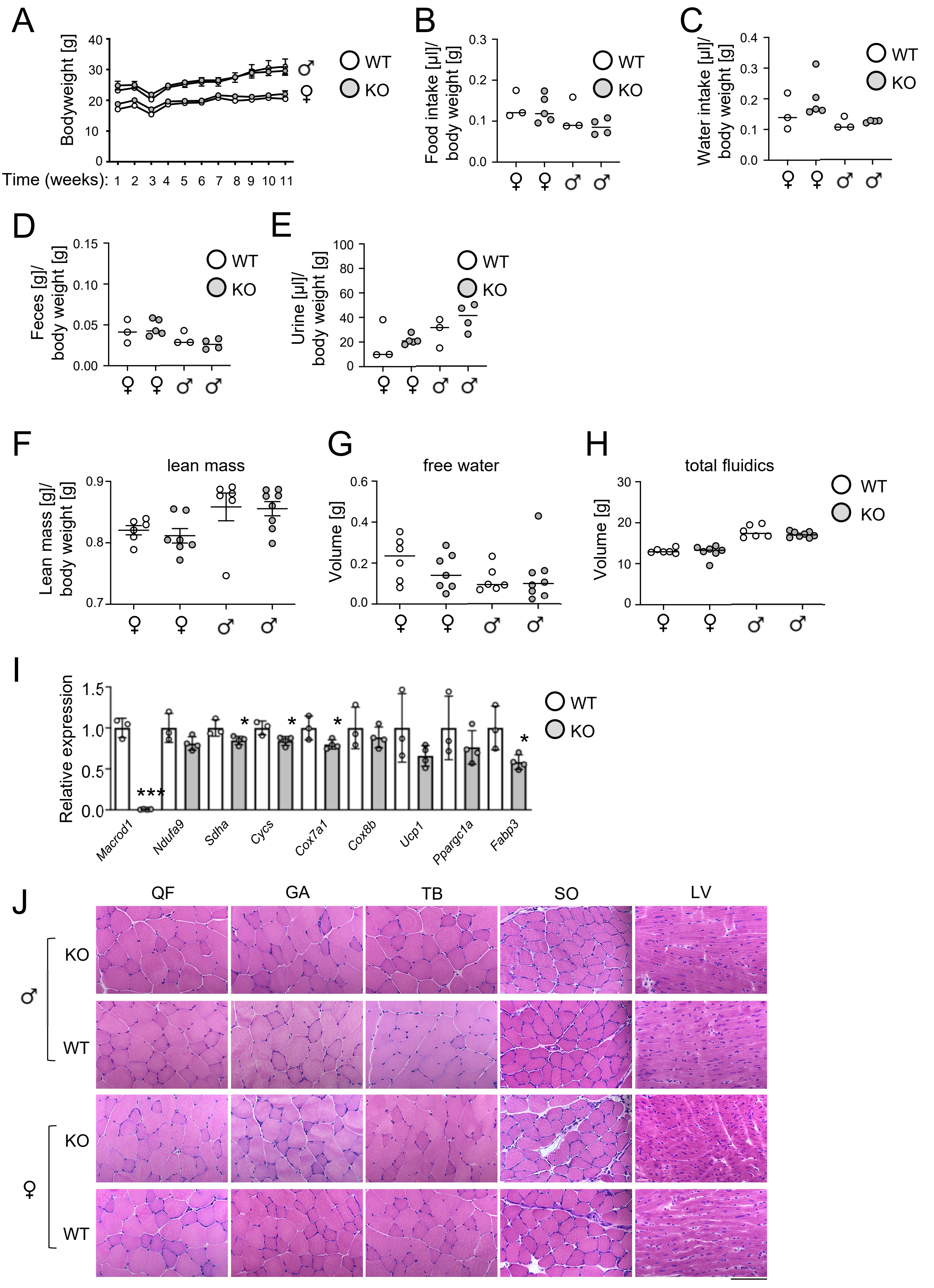

Suppl. Fig. 1: Lack of MacroD1 does not alter feeding behavior but increases body fat in mice. Male (♂) and female (♀) MacroD1$^{-/-}$ (KO) and wildtype (WT) mice were singly caged, and body weight (A), food (B), and water (C) intake as well as excretion (D and E) was monitored over 3 days. Data is shown as mean ± SD (A) or with the median depicted as black line (B-E) from n = 3 to 5 individual mice. Intake and excretion are normalized to body weight. (F-H) Body composition, including lean mass (F) and free (G) and total fluidics (H) of male (♂) and female (♀) MacroD1$^{-/-}$ (KO) and wildtype (WT) mice were analyzed via ecoMRI. Data from *n* = 6 to 8 individual mice is shown with the line indicating the median. (I) Gene expression analysis on BAT lysates from MacroD1$^{-/-}$ (KO) and wildtype (WT) mice. Data is shown as mean ± SD from *n* = 3 to 4 individual mice. Statistical analyses were performed using a two-tailed student t-test (*, p<0.05; **, p <0.005; ***, p < 0.0005) (J) HE stained sections of different muscles from MacroD1$^{-/-}$ (KO) and wildtype (WT) mice: QF: m. quadriceps femoris; GA: m. gastrocnemius; TB: m. triceps brachii; SO: m. soleus; LV: myocardium of the left ventricular free wall. Bar = 100 µm. For statistical analysis, a student t-test was performed (*n* =3-5; *, p<0.05; **, p <0.005; ***, p < 0.0005).

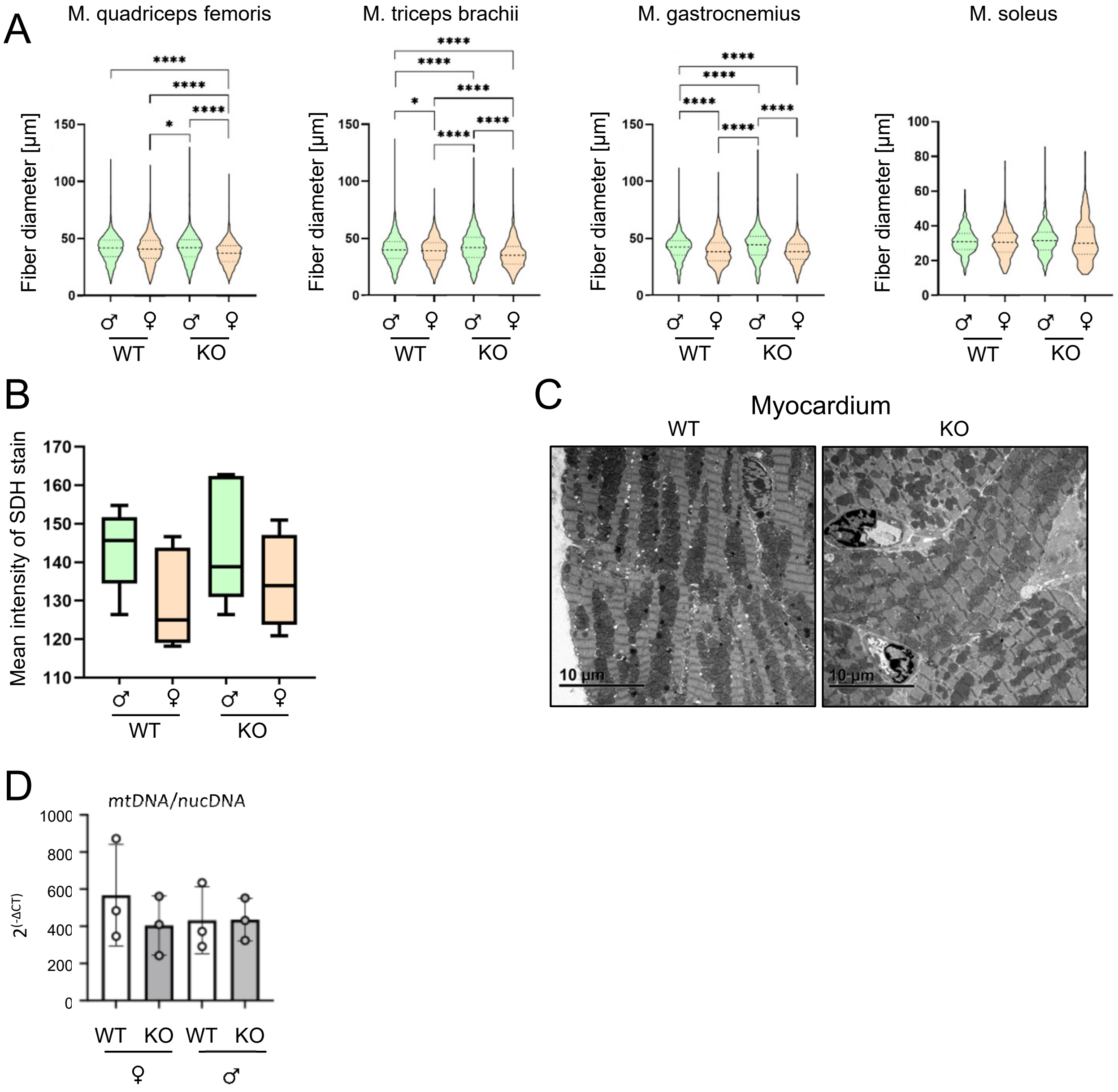
A
M. quadriceps femoris
M. triceps brachii
M. gastrocnemius
M. soleus
Fiber diameter [µm]
****
*
♂ ♀ ♂ ♀
WT
KO
B
Mean intensity of SDH stain
C
Myocardium
WT
KO
10 µm
D
mtDNA/nucDNA
2(-ΔCT)
WT KO WT KO
♀
♂

Suppl. Fig. 2: MacroD1 localizes to Mitochondria. (A) Myofiber diameter in transverse sections of HE stained cryosections of *Musculus (M.) quadriceps femoris, M. triceps brachii, M. gastrocnemius, and M. soleus* of each 10 MacroD1$^{-/-}$ (KO) and wildtype (WT) mice (each 5 males and 5 females). Median and quartiles are depicted as dashed lines. For statistical analysis, a Kruskal-Wallis H test followed by Dunn's multiple comparisons was performed (*, p<0.05; **, p <0.005; ***, p < 0.0005; ****, p < 0.0001). (B) Quantification of the *M. triceps brachii* staining intensity (arbitrary units) reflecting the mitochondrial SDH activity in MacroD1$^{-/-}$ (KO) and wildtype (WT) mice (each 5 males and 5 females). The median is depicted as straight line, whiskers indicate minimal to maximal value. For statistical analysis, a one-way ANOVA followed by Tukey's multiple comparisons was performed (*, p<0.05; **, p <0.005; ***, p < 0.0005; ****, p < 0.0001). (C) TEM images of myocardium in MacroD1$^{-/-}$ (KO) and wildtype (WT) mice. Scale bars indicate 10 µm. (D) Mitochondrial abundance was determined in the muscles of MacroD1$^{-/-}$ (KO) and wildtype (WT) male (♂) and female (♀) animals (3 animals each) by quantification of the ratio of mitochondrial (mt) to nuclear (nuc) DNA by PCR. Data is shown as mean ± SD. Statistical analyses were performed using a two-tailed student t-test (*, p<0.05; **, p <0.005; ***, p < 0.0005).

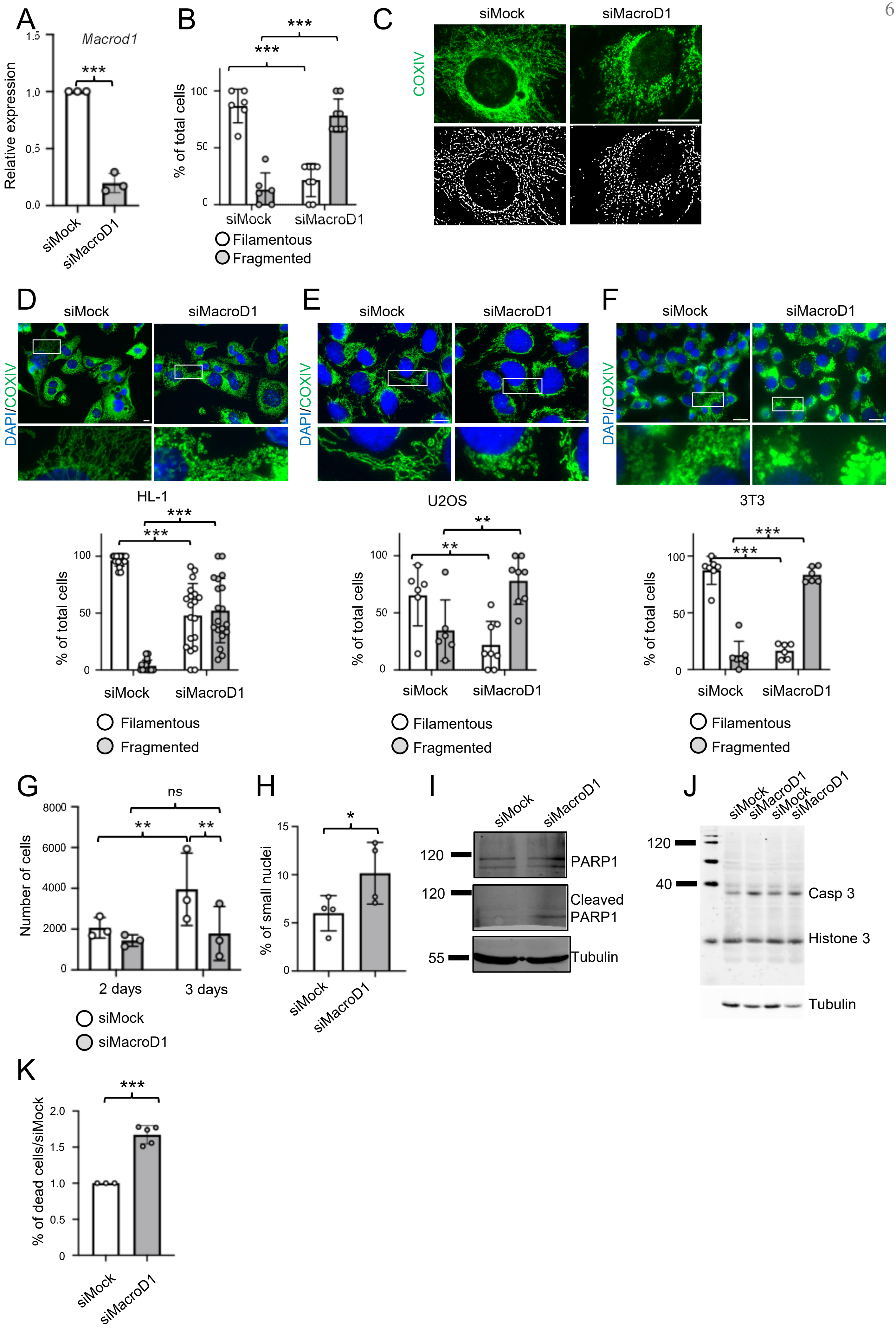

A
Macrod1
Relative expression
siMock
siMacroD1
B
% of total cells
siMock
siMacroD1
Filamentous
Fragmented
C
siMock
siMacroD1
COXIV
D
E
F
DAPI/COXIV
HL-1
U2OS
3T3
G
Number of cells
2 days
3 days
ns
H
% of small nuclei
I
PARP1
Cleaved PARP1
Tubulin
J
Casp 3
Histone 3
K
% of dead cells/siMock

Suppl. Fig. 3: Lack of MacroD1 increases mitochondrial fission and induces cell death in tested cell lines.

(A) *Macrod1* was knocked down in C2C12 cells, and knockdown efficiency was analyzed via qPCR. Data is shown as mean ± SD from 3 biological replicates. Statistical analyses were performed using a two-tailed student t-test (*, $p<0.05$; **, $p<0.005$; ***, $p < 0.0005$). (B) *Macrod1* was knocked down for two days in C2C12 cells, and mitochondrial morphology was analyzed manually by IF. Data is shown as mean ± SD from up to 8 individual cells. Statistical analyses were performed using a two-tailed student t-test (*, $p<0.05$; **, $p<0.005$; ***, $p < 0.0005$). (C) For automated, quantitative analysis of the same images, mitochondrial signals were skeletonized using the Mitochondrial Analyzer FIJI plugin (black-and-white pictures). Scale bar indicates 10 µm. (D-F) The gene of MacroD1 was knocked down for 2 days in HL1 (D), U2OS (E), or 3T3 cells (F), respectively, and mitochondrial morphology was analyzed via IF. Data is shown as mean ± SD from up to 9 individual cells. Statistical analyses were performed using a two-tailed student t-test (*, $p<0.05$; **, $p<0.005$; ***, $p < 0.0005$). (G and H) Two and three days after siRNA knockdown of MacroD1 in C2C12, total cell numbers (G) and nuclear sizes (H) were assessed via QIBC [1,2]. Data is shown as mean ± SD from 4 individual experiments. Statistical analyses were performed using a two-tailed student t-test (*, $p<0.05$; **, $p<0.005$; ***, $p < 0.0005$). (I and J) *Macrod1* was knocked down for 2 days in C2C12 cells, and PARP1 cleavage and caspase 3 (Casp 3) level were analyzed by Western blot. (K) Three days after the knockdown of *Macrod1* in HL1 cells, viability was assessed via AnnexinV staining and subsequent flow cytometry. Percentages were normalized to value of siMock. Data is shown as mean ± SD from 3 biological replicates. Statistical analyses were performed using a two-tailed student t-test (*, $p<0.05$; **, $p<0.005$; ***, $p < 0.0005$). Scale bars indicate 10 µm.

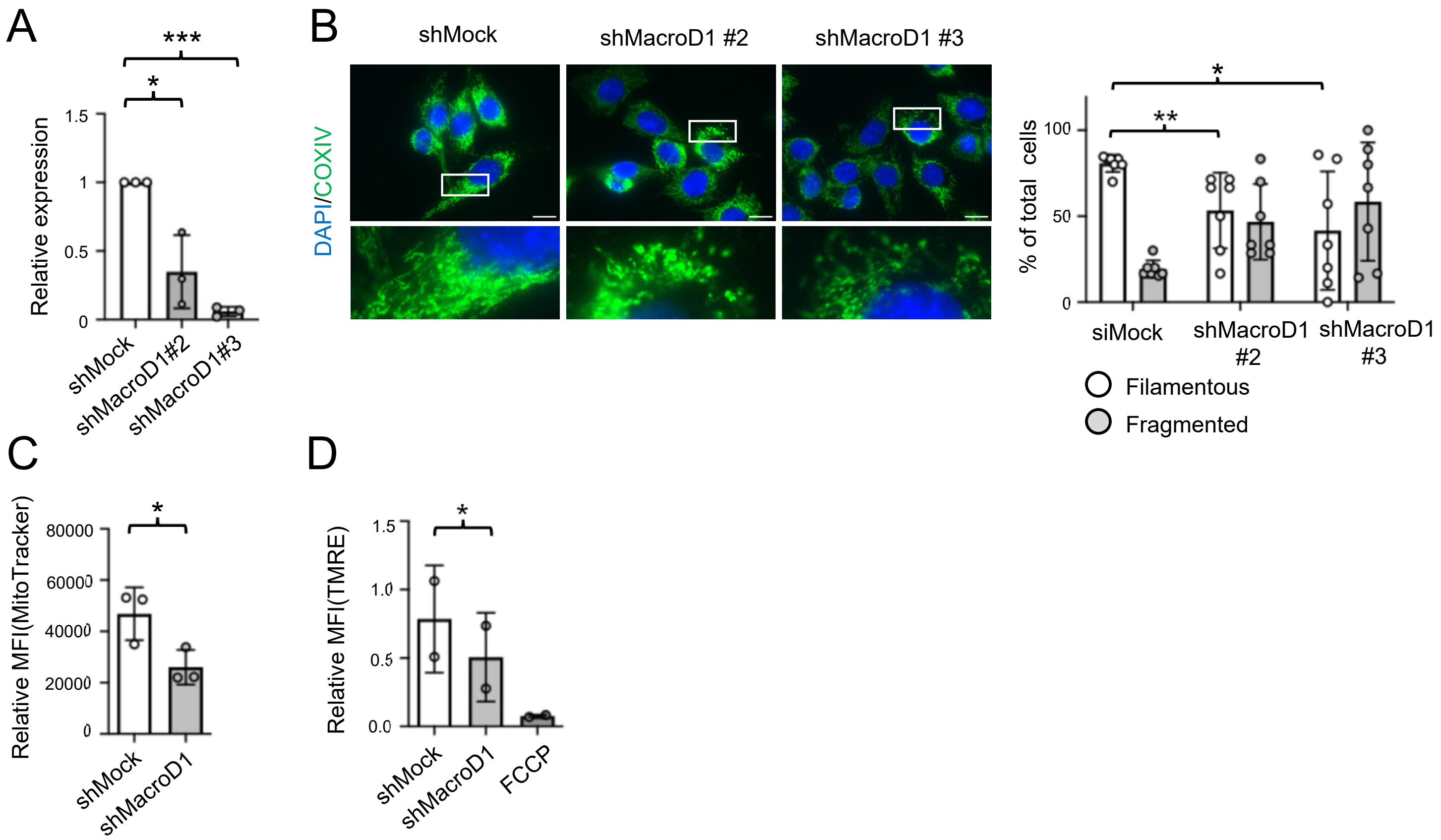
A
***
*
Relative expression
1.5
1
0.5
0
shMock
shMacroD1#2
shMacroD1#3
B
shMock
shMacroD1 #2
shMacroD1 #3
DAPI/COXIV
*
**
% of total cells
100
50
0
siMock
shMacroD1 #2
shMacroD1 #3
Filamentous
Fragmented
C
*
Relative MFI(MitoTracker)
80000
60000
40000
20000
0
shMock
shMacroD1
D
*
Relative MFI(TMRE)
1.5
1.0
0.5
0.0
shMock
shMacroD1
FCCP

Suppl. Fig. 4: Lack of MacroD1 impairs mitochondrial morphology and function. (A) *Macrod1* was stably knocked down in C2C12 cells via shRNA transduction and verified by qPCR. Data is shown as mean ± SD from 3 biological replicates. Statistical analyses were performed using a two-tailed student t-test (*, $p<0.05$; **, $p<0.005$; ***, $p<0.0005$). (B) The same cells were fixed, stained with an anti-COXIV antibody and mitochondrial morphology was analyzed manually by fluorescent microscopy. Scale bars indicate 10 µm. Data is shown as mean ± SD from up to 7 individual cells. Statistical analyses were performed using a two-tailed student t-test (*, $p<0.05$; **, $p<0.005$; ***, $p<0.0005$). (C-D) Stable *Macrod1* knockdown C2C12 cells were stained with MitoTracker (C) or TMRE (D) and mitochondrial load (C) as well as polarization (D) were analyzed by flow cytometry. Data is shown as mean ± SD from 3 biological replicates. Statistical analyses were performed using a two-tailed student t-test (*, $p<0.05$; **, $p<0.005$; ***, $p<0.0005$).

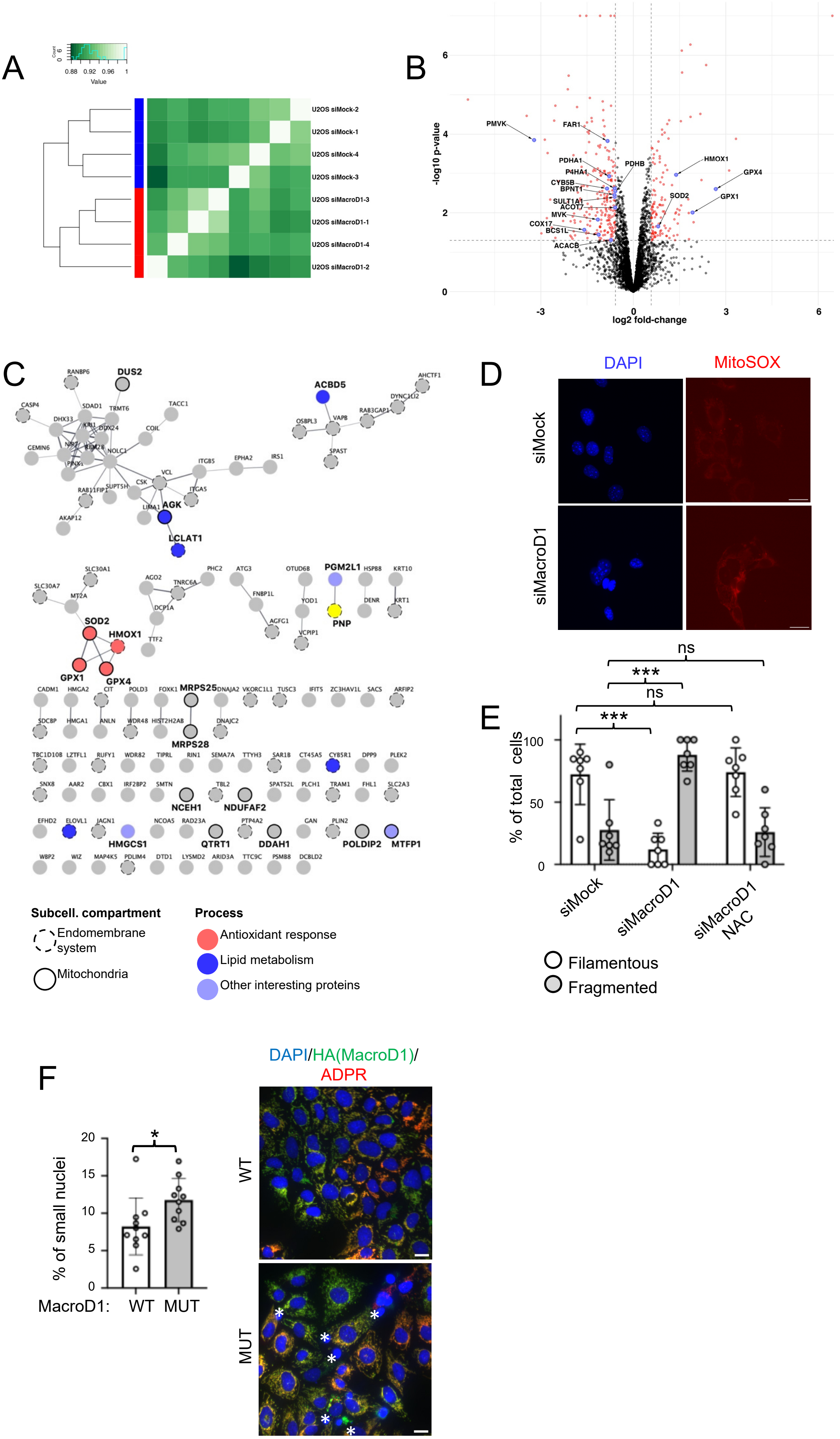
A
Count
0.88 0.92 0.96 1
Value
U2OS siMock-2
U2OS siMock-1
U2OS siMock-4
U2OS siMock-3
U2OS siMacroD1-3
U2OS siMacroD1-1
U2OS siMacroD1-4
U2OS siMacroD1-2
B
-log10 p-value
log2 fold-change
PMVK
FAR1
PDHA1
P4HA1
PDHB
CYB5B
BPNT1
SULT1A1
ACOT7
MVK
COX17
BCS1L
ACACB
HMOX1
GPX4
SOD2
GPX1
C
DUS2
ACBD5
AGK
LCLAT1
PGM2L1
PNP
SOD2
HMOX1
GPX1
GPX4
MRPS25
MRPS28
NCEH1
NDUFAF2
HMGCS1
QTRT1
DDAH1
POLDIP2
MTFP1
Subcell. compartment
Endomembrane system
Mitochondria
Process
Antioxidant response
Lipid metabolism
Other interesting proteins
D
DAPI
MitoSOX
siMock
siMacroD1
E
ns
***
ns
***
% of total cells
100
50
0
siMock
siMacroD1
siMacroD1 NAC
Filamentous
Fragmented
F
DAPI/HA(MacroD1)/ADPR
% of small nuclei
20
15
10
5
0
*
MacroD1: WT MUT
WT
MUT

Suppl. Fig. 5: MacroD1 gene knockdown alters the abundance of various metabolic proteins. (A and B) *MACROD1* was knocked down for 2 days in U2OS cells and the whole proteome was analyzed via LFQ-based mass spectrometry. Unsupervised hierarchical cluster analysis of all replicates measured (blue group: siMock, red group: siMacroD1) (A) and volcano plot with all differentially regulated proteins either marked in red (any differentially regulated protein) or blue (highlighted differentially regulated protein) (B). (C) STRING analysis of all significantly up-regulated proteins. (D) Stable *Macrod1* knockdown C2C12 cells were stained with MitoSOX and images were acquired by fluorescence microscopy. (E) *Macrod1* was knocked down in C2C12 cells, 24h after siRNA transfection, cells were treated with the antioxidant NAC or left untreated, and another 24h later, cells were fixed, mitochondria were stained with an anti-COXIV antibody and mitochondrial morphology was manually assessed. Data is shown as mean ± SD from up to 7 individual cells. Statistical analyses were performed using a two-tailed student t-test (*, $p<0.05$; **, $p<0.005$; ***, $p < 0.0005$). (F) Endogenous *MACROD1* was knocked down from U2OS cells stably overexpressing either wildtype (WT) or mutant (MUT) MacroD1 and nuclear size was analyzed by IF after fixation and staining. Quantifications are shown on the left and representative images on the right. Small nuclei are marked with a white asterix. Scale bars indicate 10 µm. Data is shown as mean ± SD from all cells analyzed in up to 10 individual images. Statistical analyses were performed using a two-tailed student t-test (*, $p<0.05$; **, $p<0.005$; ***, $p < 0.0005$).

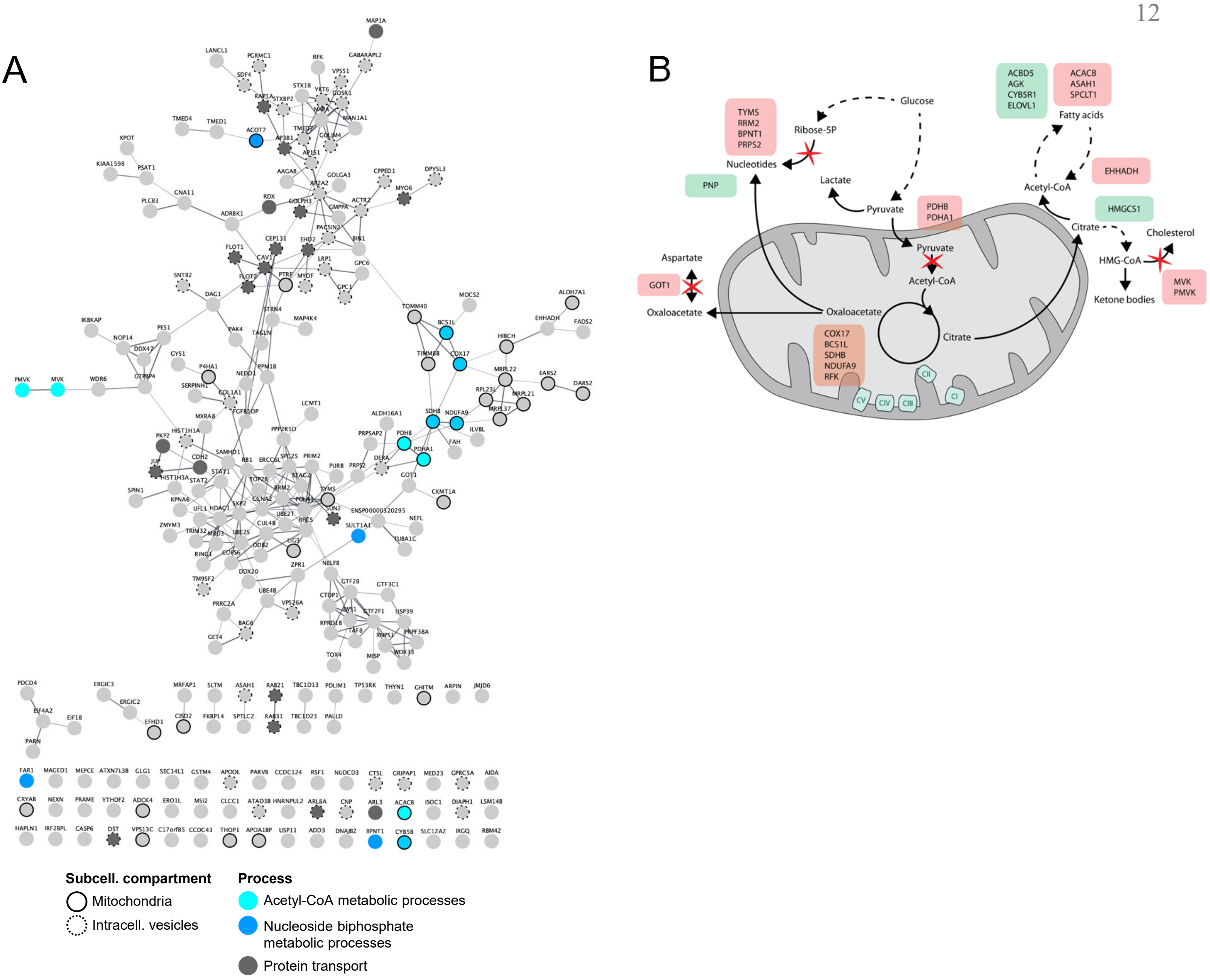
A
B
Subcell. compartment
Mitochondria
Intracell. vesicles
Process
Acetyl-CoA metabolic processes
Nucleoside biphosphate metabolic processes
Protein transport
Glucose
Ribose-5P
TYMS
RRM2
BPNT1
PRPS2
Nucleotides
PNP
Lactate
Pyruvate
PDHB
PDHA1
Acetyl-CoA
Aspartate
GOT1
Oxaloacetate
Citrate
COX17
BCS1L
SDHB
NDUFA9
RFK
CI
CII
CIII
CIV
CV
ACBD5
AGK
CYB5R1
ELOVL1
ACACB
ASAH1
SPCLT1
Fatty acids
EHHADH
HMGCS1
Cholesterol
HMG-CoA
MVK
PMVK
Ketone bodies

Suppl. Fig. 6: Knockdown of *MACROD1* decreases the abundance of mitochondrial proteins involved in OXPHOS

(A) STRING analysis of all significantly down-regulated proteins. (B) Overview on the overall changes in the abundance of metabolic proteins following *MACROD1* knockdown.

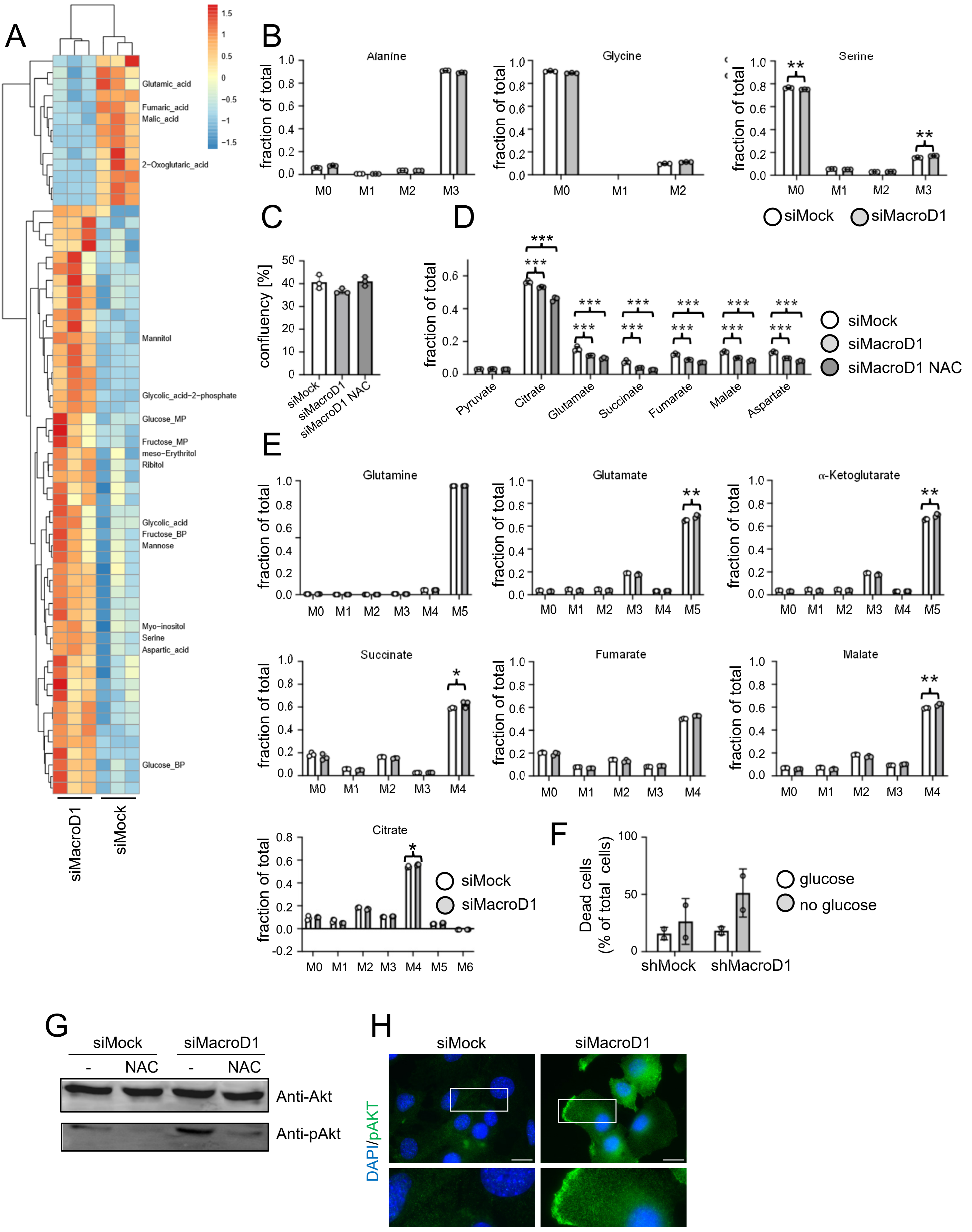
A
Glutamic_acid
Fumaric_acid
Malic_acid
2-Oxoglutaric_acid
Mannitol
Glycolic_acid-2-phosphate
Glucose_MP
Fructose_MP
meso-Erythritol
Ribitol
Glycolic_acid
Fructose_BP
Mannose
Myo-inositol
Serine
Aspartic_acid
Glucose_BP
siMacroD1
siMock
B
Alanine
Glycine
Serine
fraction of total
siMock
siMacroD1
C
confluency [%]
siMock
siMacroD1
siMacroD1 NAC
D
fraction of total
Pyruvate
Citrate
Glutamate
Succinate
Fumarate
Malate
Aspartate
siMock
siMacroD1
siMacroD1 NAC
E
Glutamine
Glutamate
α-Ketoglutarate
Succinate
Fumarate
Malate
Citrate
fraction of total
siMock
siMacroD1
F
Dead cells (% of total cells)
shMock
shMacroD1
glucose
no glucose
G
siMock
siMacroD1
-
NAC
Anti-Akt
Anti-pAkt
H
siMock
siMacroD1
DAPI/pAKT

Suppl. Fig. 7: Knockdown of *Macrod1* alters the cells' metabolic profile.

(A) Untargeted metabolome analysis of siMacroD1 and siMock treated C2C12 cells 60h after siRNA transfection. (B-E) C2C12 cells transfected with siMacroD1 or a scrambled siRNA (siMock) were incubated in presence of either U-^{13}C glucose (B-D) or U-^{13}C glutamine (E) and targeted metabolome analysis was performed. Data is shown as mean ± SD from 3 biological replicates. Statistical analyses were performed using a two-tailed student t-test (*, p<0.05; **, p <0.005; ***, p < 0.0005). (F) shMacroD1 and shMock C2C12 cells were subjected to glucose starvation and cell viability was analyzed via flow cytometry. Data is shown as mean ± SD from 2 biological replicates. Statistical analyses were performed using a two-tailed student t-test (*, p<0.05; **, p <0.005; ***, p < 0.0005). *Macrod1* was knocked down in C2C12 cells and Akt phosphorylation was assessed via WB (G) or IF (H). Scale bars indicate 10 µm.

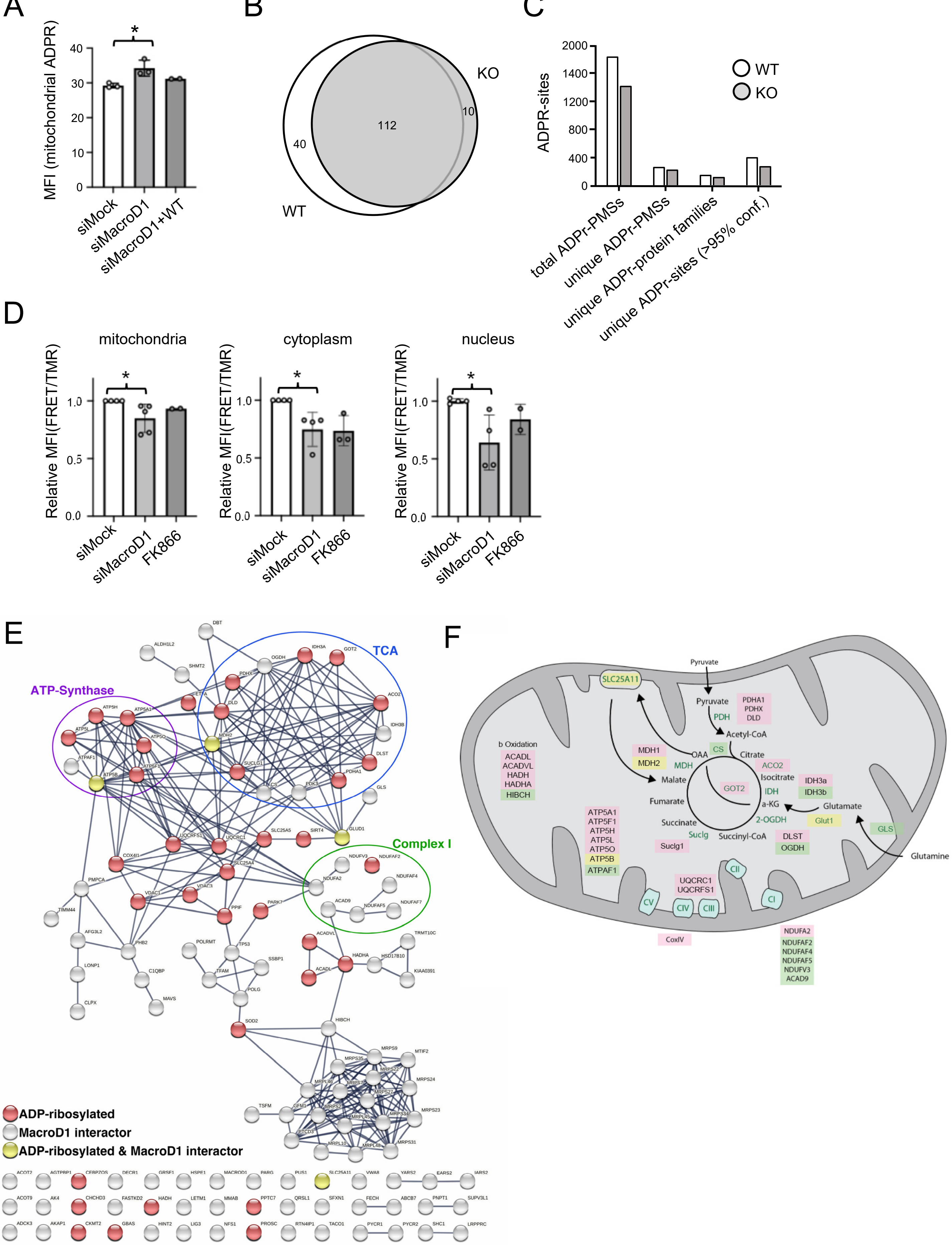
A
MFI (mitochondrial ADPR)
40
30
20
10
0
*
siMock
siMacroD1
siMacroD1+WT
B
WT
KO
40
112
10
C
ADPR-sites
2000
1400
1200
800
400
0
WT
KO
total ADPr-PMSs
unique ADPr-PMSs
unique ADPr-protein families
unique ADPr-sites (>95% conf.)
D
mitochondria
cytoplasm
nucleus
Relative MFI(FRET/TMR)
1.0
0.5
0.0
*
siMock
siMacroD1
FK866
E
ATP-Synthase
TCA
Complex I
ADP-ribosylated
MacroD1 interactor
ADP-ribosylated & MacroD1 interactor
F
Pyruvate
SLC25A11
PDHA1
PDHX
DLD
PDH
Acetyl-CoA
b Oxidation
ACADL
ACADVL
HADH
HADHA
HIBCH
MDH1
MDH2
MDH
OAA
CS
Citrate
ACO2
Isocitrate
IDH3a
IDH3b
IDH
Malate
GOT2
a-KG
Glutamate
Glut1
GLS
Glutamine
Fumarate
2-OGDH
Succinate
Suclg
Succinyl-CoA
DLST
OGDH
Suclg1
ATP5A1
ATP5F1
ATP5H
ATP5L
ATP5O
ATP5B
ATPAF1
UQCRC1
UQCRFS1
CII
CV
CIV
CIII
CI
CoxIV
NDUFA2
NDUFAF2
NDUFAF4
NDUFAF5
NDUFV3
ACAD9

Suppl. Fig. 8: Knockdown of *Macrod1* influences mitochondrial ADP-ribosylation.

(A) Endogenous *Macrod1* was knocked down in presence or absence of overexpressed MacroD1 in C2C12 cells and 3 days after knockdown, mitochondrial ADP-ribosylation was analyzed by IF. Data is shown as mean ± SD from 2 to 3 biological replicates. Statistical analyses were performed using a two-tailed student t-test (*, p<0.05; **, p <0.005; ***, p < 0.0005). (B) The ADP-ribosylome of mouse muscle tissues for wildtype (WT) and MacroD1$^{-/-}$ (KO) were identified using mass spectrometry analyses. (C) Qualitative comparisons were made of all ADP-ribosylated peptide spectral matches (PSMs), unique peptides, unique proteins and unique confidently identified ADPr-acceptor sites between WT and MacroD1 KO tissues. (D) *MACROD1* was knocked down in Stable U2OS Flp-In™ T-Rex™ cells expressing inducible NAD$^+$ sensors and mitochondrial, cytoplasmic and nuclear NAD$^+$ level was assessed by flow cytometry. The NAMPT inhibitor F866 was used as positive control. Data is shown as mean ± SD from 2 to 4 biological replicates. Statistical analyses were performed using a two-tailed student t-test (*, p<0.05; **, p <0.005; ***, p < 0.0005). (E) STRING analysis of the comparison between the ADP-ribosylome of mouse muscle and the MacroD1 interactome [3]. ADP-ribosylated proteins in red, interactor in grey, and ADP-ribosylated interactors in yellow. (F) Schematic localization of groups from (E). ADP-ribosylated proteins in red, interactor in green, and ADP-ribosylated interactors in yellow.

References in Supplementary Information

1. Toledo, L. I. et al. ATR prohibits replication catastrophe by preventing global exhaustion of RPA. Cell 155, 1088-1103, doi:10.1016/j.cell.2013.10.043 (2013).
2. Michelena, J. et al. Analysis of PARP inhibitor toxicity by multidimensional fluorescence microscopy reveals mechanisms of sensitivity and resistance. Nat Commun 9, 2678, doi:10.1038/s41467-018-05031-9 (2018).
3. Zaja, R. et al. Comparative analysis of MACROD1, MACROD2 and TARG1 expression, localisation and interactome. Sci Rep 10, 8286, doi:10.1038/s41598-020-64623-y (2020).

SOURCE DATA
Uncropped scans of Supplementary Data

Supplementary Figure 3I

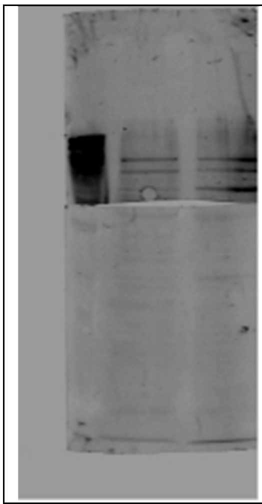	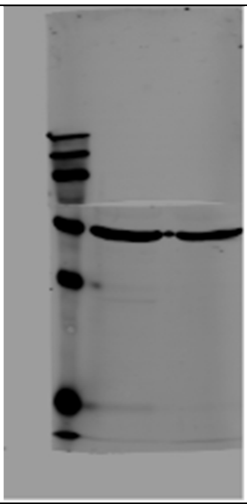	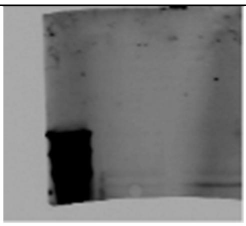
PARP1	Tubulin	Cleaved PARP1

Supplementary Figure 7G

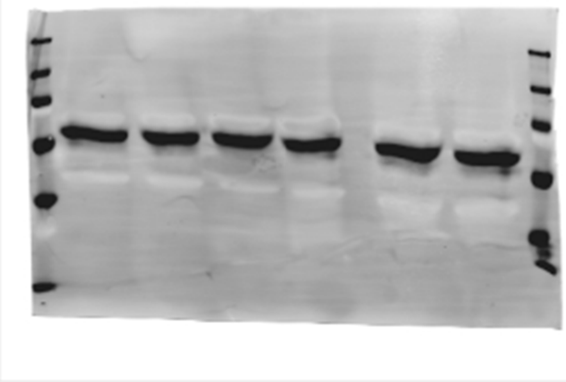	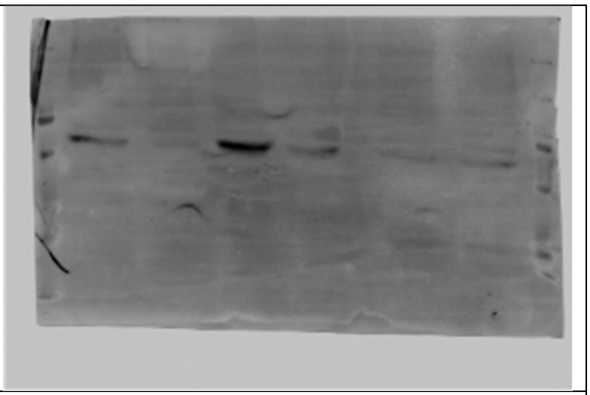
Akt	pAkt

Suppl. Information Gating: Gating strategies of flow cytometry experiments.

(see next page)

(A) Gating strategy for all dead cell experiments, displayed in Figures 3C, 3F, 4E, S3K, S7D.

(B) Gating strategy for Fig. 3E.

(C) Gating strategy for Suppl. Fig. 4C.

(D) Gating strategy for Suppl. Fig. 4D.

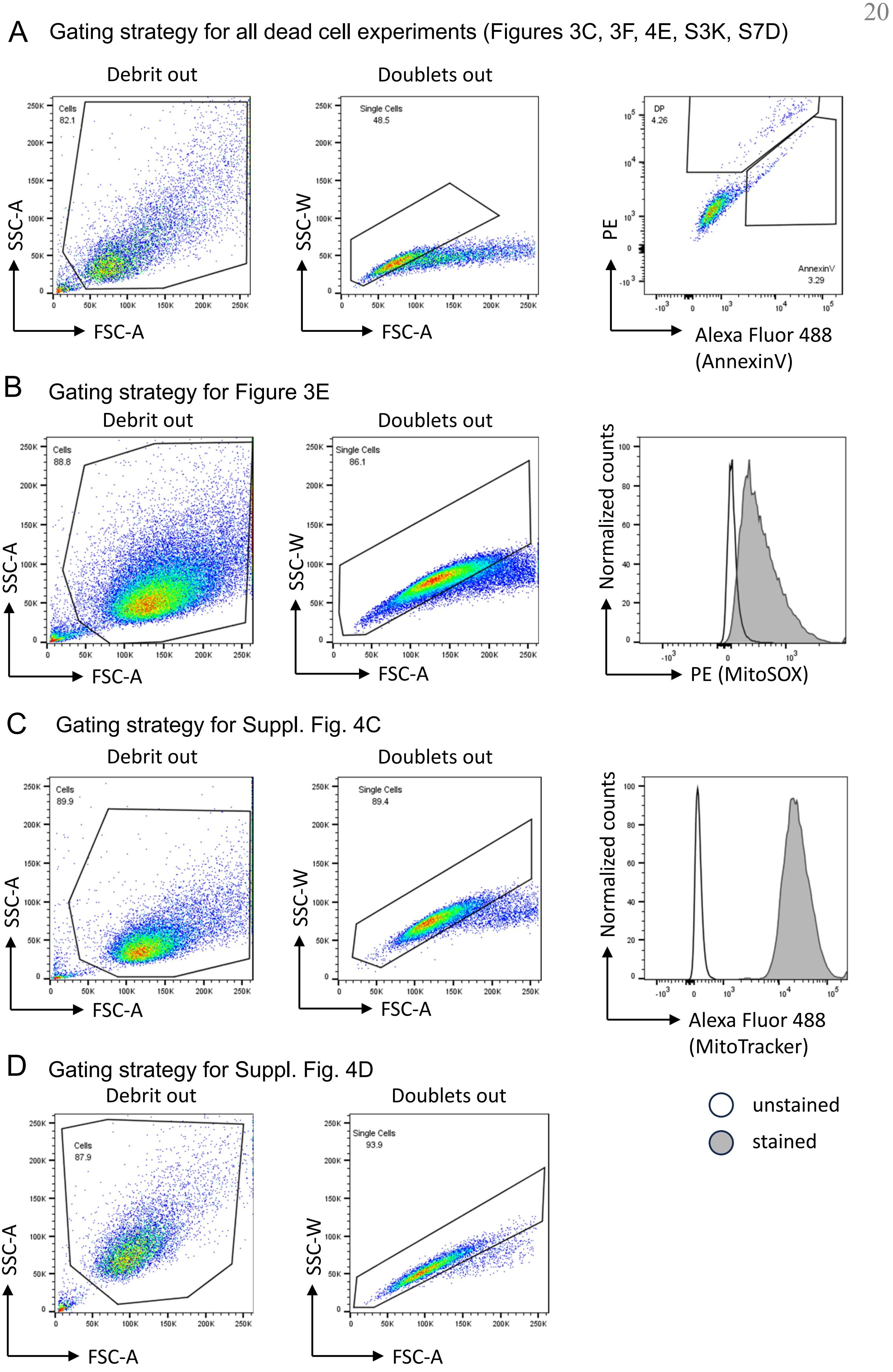
A Gating strategy for all dead cell experiments (Figures 3C, 3F, 4E, S3K, S7D)
Debrit out
Doublets out
Cells
82.1
Single Cells
48.5
DP
4.26
AnnexinV
3.29
SSC-A
SSC-W
PE
FSC-A
FSC-A
Alexa Fluor 488
(AnnexinV)
B Gating strategy for Figure 3E
Debrit out
Doublets out
Cells
88.8
Single Cells
86.1
SSC-A
SSC-W
Normalized counts
FSC-A
FSC-A
PE (MitoSOX)
C Gating strategy for Suppl. Fig. 4C
Debrit out
Doublets out
Cells
89.9
Single Cells
89.4
SSC-A
SSC-W
Normalized counts
FSC-A
FSC-A
Alexa Fluor 488
(MitoTracker)
D Gating strategy for Suppl. Fig. 4D
Debrit out
Doublets out
Cells
87.9
Single Cells
93.9
SSC-A
SSC-W
FSC-A
FSC-A
unstained
stained
