# Supplementary material for: MacroD1 sustains mitochondrial integrity and oxidative metabolism
Source: Nat Commun. 2025 Aug 15;16:7595. doi: 10.1038/s41467-025-62410-9 (PMC12356970; doi:10.1038/s41467-025-62410-9)
Supplement: Supplementary file 2 — Description of Additional Supplementary Files [file 41467_2025_62410_MOESM2_ESM.pdf]

Description of Additional Supplementary Files

Supplementary Data 1: siMock-U2OS versus siMacroD1-U2OS LFQ proteome analyses.

Supplementary Data 2: Wildtype and MacroD1 knockout Mouse Muscle ADP-ribosylomes Identified via Mass-Spectrometry.

Supplementary Data 3: Wildtype and MacroD1 knockout Mouse Muscle LFQ proteome analyses.
