# Supplementary material for: MacroD1 sustains mitochondrial integrity and oxidative metabolism
Source: Nat Commun. 2025 Aug 15;16:7595. doi: 10.1038/s41467-025-62410-9 (PMC12356970; doi:10.1038/s41467-025-62410-9)
Supplement: Supplementary file 6 — Reporting Summary [file 41467_2025_62410_MOESM6_ESM.pdf]

Corresponding author(s): Michael O. Hottiger

Last updated by author(s): Apr 17, 2025

Reporting Summary

Nature Portfolio wishes to improve the reproducibility of the work that we publish. This form provides structure for consistency and transparency in reporting. For further information on Nature Portfolio policies, see our Editorial Policies and the Editorial Policy Checklist.

Statistics

For all statistical analyses, confirm that the following items are present in the figure legend, table legend, main text, or Methods section.

n/a	Confirmed	
☐	☒	The exact sample size (*n*) for each experimental group/condition, given as a discrete number and unit of measurement
☐	☒	A statement on whether measurements were taken from distinct samples or whether the same sample was measured repeatedly
☐	☒	The statistical test(s) used AND whether they are one- or two-sided<br>*Only common tests should be described solely by name; describe more complex techniques in the Methods section.*
☒	☐	A description of all covariates tested
☐	☒	A description of any assumptions or corrections, such as tests of normality and adjustment for multiple comparisons
☐	☒	A full description of the statistical parameters including central tendency (e.g. means) or other basic estimates (e.g. regression coefficient) AND variation (e.g. standard deviation) or associated estimates of uncertainty (e.g. confidence intervals)
☐	☒	For null hypothesis testing, the test statistic (e.g. *F*, *t*, *r*) with confidence intervals, effect sizes, degrees of freedom and *P* value noted<br>*Give P values as exact values whenever suitable.*
☒	☐	For Bayesian analysis, information on the choice of priors and Markov chain Monte Carlo settings
☒	☐	For hierarchical and complex designs, identification of the appropriate level for tests and full reporting of outcomes
☒	☐	Estimates of effect sizes (e.g. Cohen's *d*, Pearson's *r*), indicating how they were calculated

Our web collection on statistics for biologists contains articles on many of the points above.

Software and code

Policy information about availability of computer code

Data collection	Olympus ScanR Image Analysis Software version 3.0.1; Gatan Microscopical Suite, Digital Micrograph, Version 230.540; MetaboliteDetector software package for GC-MS chromatograms; MassHunter 10.0 software (Agilent Technologies) metabolite MS; Visiopharm (Version 2023.01.3.14018; Visiopharm, Hoersholm, Denmark) served for the morphometry of muscle fibers;
Data analysis	Olympus ScanR Image Analysis Software version 3.0.1; MetaboliteDetector software package for GC-MS chromatograms;MassHunter 10.0 software (Agilent Technologies) metabolite MS; Visiopharm (Version 2023.01.3.14018; Visiopharm, Hoersholm, Denmark) served for the morphometric analyses of muscle fibers; MaxQuant (version 1.6.2.3); regular statistical analysis was performed with Prism version 9.5.1.

For manuscripts utilizing custom algorithms or software that are central to the research but not yet described in published literature, software must be made available to editors and reviewers. We strongly encourage code deposition in a community repository (e.g. GitHub). See the Nature Portfolio guidelines for submitting code & software for further information.

Data

Policy information about availability of data

All manuscripts must include a data availability statement. This statement should provide the following information, where applicable:

- Accession codes, unique identifiers, or web links for publicly available datasets
- A description of any restrictions on data availability
- For clinical datasets or third party data, please ensure that the statement adheres to our policy

The data that support the findings of this study are available in the main text, the supplementary materials and from the corresponding author upon reasonable request. Source data are provided with this paper. The mass spectrometry data used in this study have been deposited to the ProteomeXchange Consortium via the PRIDE (http://www.ebi.ac.uk/pride) partner repository with the data set identifier PXD020281 (https://www.ebi.ac.uk/pride/archive/projects/PXD020281).

Research involving human participants, their data, or biological material

Policy information about studies with human participants or human data. See also policy information about sex, gender (identity/presentation), and sexual orientation and race, ethnicity and racism.

Reporting on sex and gender	N/A
Reporting on race, ethnicity, or other socially relevant groupings	N/A
Population characteristics	N/A
Recruitment	N/A
Ethics oversight	N/A

Note that full information on the approval of the study protocol must also be provided in the manuscript.

Field-specific reporting

Please select the one below that is the best fit for your research. If you are not sure, read the appropriate sections before making your selection.

☒ Life sciences ☐ Behavioural & social sciences ☐ Ecological, evolutionary & environmental sciences

For a reference copy of the document with all sections, see nature.com/documents/nr-reporting-summary-flat.pdf

Life sciences study design

All studies must disclose on these points even when the disclosure is negative.

Sample size	No samples size calculation was performed. Cellular analyses are based on large numbers of cells. Analyses of animals are based on experience and responsible and ethical use of animals.
Data exclusions	No data was excluded.
Replication	Experiments were replicated as stated in the text.
Randomization	Animals were randomly allocated into groups by one researcher and then analyzed by a blinded other researcher.
Blinding	Investigators performing analyses were blinded to the group allocation.

Reporting for specific materials, systems and methods

We require information from authors about some types of materials, experimental systems and methods used in many studies. Here, indicate whether each material, system or method listed is relevant to your study. If you are not sure if a list item applies to your research, read the appropriate section before selecting a response.

Materials & experimental systems

n/a	Involved in the study
☐	☒ Antibodies
☐	☒ Eukaryotic cell lines
☒	☐ Palaeontology and archaeology
☐	☒ Animals and other organisms
☒	☐ Clinical data
☒	☐ Dual use research of concern
☒	☐ Plants

Methods

n/a	Involved in the study
☒	☐ ChIP-seq
☐	☒ Flow cytometry
☒	☐ MRI-based neuroimaging

Antibodies

Antibodies used	Cox IV (Abcam polyclonal ab16056)<br>MyHC-7 (clone NOQ7.5.4D)<br>ATP5a (Abcam clone 15H4C4)<br>anti-HA tag (Biolegend polyclonal Poly9023, previously Covance)<br>rabbit anti-ADPR (Hottiger laboratory)
Validation	CoxIV antibody was validate in WB onlysates of different species and tissues and produced bands of appropriate size. Furthermore, the CoxIV antibody was used in immunohistochemistry and immunofluorescence stainings on cells and tissues and produced the appropriate mitochondrial staining patterns (data from Abcam).<br>Anti-MyHC-7 clone NOQ7.5.4D is a videly used established monoclonal antibody that was validated in WB on rat and mouse skeletal and heart muscle tissue while it did not produce bands with smooth muscle, brain and liver tissue of rats or mice (data from ThermoFisher).<br>ATP5a (clone 15H4C4) is an antibody clone that is widely used as mitochondrial marker. It has been used in immunofluorescence on mitochondria-rich spermatids, collocalizes with Mitotracker dye, reveals specific bands in WB, and is mutually exclusive witha tubulin staining (data from Abcam).<br>Our polyclonal anti-ADPR antibody was described in numerous papers: PMIDs 37587695, 36515832, 34060040, 33808662, 33742140, and 33450210.

Eukaryotic cell lines

Policy information about cell lines and Sex and Gender in Research

Cell line source(s)	C2C12 (CRL-1772), U2OS (HTB-96), HEK293T (CRL-3216), NIH-3T3 (CRL-1658) were obtained from ATCC, and HL-1 was obtained from Millipore (SCC065).
Authentication	Cell lines were not re-authenticated but were morphologically and functionally identical to the original description.
Mycoplasma contamination	All cell lines were regularly tested for Mycoplasma and used only if negatively tested.
Commonly misidentified lines<br>(See ICLAC register)	N/A

Animals and other research organisms

Policy information about studies involving animals; ARRIVE guidelines recommended for reporting animal research, and Sex and Gender in Research

Laboratory animals	Mice of strain C57BL6/N-MacroD1<em2Wtsi> were obtained from the Wellcome Trust Sanger Institute (Hinxton, UK) and used with the appropriate WT control animals obtained from Janvier. The animals were used at 6-7 weeks of age.
Wild animals	N/A
Reporting on sex	Sex is reported. Studies were designed to included both sexes. Retrospective analysis revealed that the main findings were not sex-dependent, while certain differences (fiber diameter) were significant only in one sex.
Field-collected samples	N/A
Ethics oversight	Animal studies were approved by the Cantonal Ethics Committee for Animal Research of the Canton of Zurich, Switzerland.

Note that full information on the approval of the study protocol must also be provided in the manuscript.

Plants

Seed stocks: N/A

Novel plant genotypes: N/A

Authentication: N/A

Flow Cytometry

Plots

Confirm that:

☒ The axis labels state the marker and fluorochrome used (e.g. CD4-FITC).

☒ The axis scales are clearly visible. Include numbers along axes only for bottom left plot of group (a 'group' is an analysis of identical markers).

☒ All plots are contour plots with outliers or pseudocolor plots.

☒ A numerical value for number of cells or percentage (with statistics) is provided.

Methodology

Sample preparation: Only cell lines were analyzed by FC.

Instrument: LSR II Fortessa

Software: Acquisition was done with standard BD bioscience acquisiton software. Analysis was done with FloJo v10.

Cell population abundance: N/A

Gating strategy: All gating strategies are displayed in Suppl. Fig 9. For determination of dead/apoptotic cells, DNA dyes and Annexin-V staining were used which produce clearly distinct stained populations which were then quantified. For all other analyses, MFI of the whole FSC/SSC-singlet gated population were determined without pre-selection of positive or negative fractions.

☒ Tick this box to confirm that a figure exemplifying the gating strategy is provided in the Supplementary Information.
